# The effect of combining therapeutic drug monitoring of antihypertensive drugs with personalised feedback on adherence and resistant hypertension: the (RHYME-RCT) trial protocol of a multi-centre randomised controlled trial

**DOI:** 10.1186/s12872-023-03114-0

**Published:** 2023-02-14

**Authors:** L. E. J. Peeters, M. H. W. Kappers, E. Boersma, E. K. Massey, L. van Dijk, T. van Gelder, B. C. P. Koch, J. Versmissen

**Affiliations:** 1 Department of Internal Medicine, Erasmus MC, University Medical Centre Rotterdam, Rotterdam, The Netherlands; 2 Department of Hospital Pharmacy, Erasmus MC, Erasmus University Medical Centre, Rotterdam, The Netherlands; 3 Department of Internal Medicine, Amphia Hospital, Breda, The Netherlands; 4 Department of Cardiology, Erasmus MC, Erasmus University Medical Centre, Rotterdam, The Netherlands; 5 Department Pharmaceutical Care, Nivel, Netherlands Institute for Health Services Research, Utrecht, The Netherlands; 6 Unit of PharmacoTherapy, Epidemiology and Economics, Groningen Research Institute of Pharmacy, University of Groningen, Groningen, The Netherlands

**Keywords:** Hypertension, Therapeutic drug monitoring, Intervention, Adherence, Antihypertensive drugs, Blood pressure

## Abstract

**Background:**

Adherence to antihypertensive drugs (AHDs) is important for adequate blood pressure control. Not taking these drugs as prescribed is one of the main underlying causes for resistant hypertension (RH), which in turn leads to an increased risk of cardiovascular events, stroke and kidney damage. Therefore, correct identification of patients that are non-adherent to AHDs is crucial to improve clinical outcome. For this goal, therapeutic drug monitoring is the most reliable method. The primary objective of this trial is to investigate whether monitoring of drug concentrations with a dried blood spot (DBS) sampling method combined with personalised feedback leads to a decrease in prevalence of RH after 12 months due to an increase in adherence. Secondary objectives include the difference over time in the number of required AHDs as well as the defined daily dose (DDD). Lastly, the cost-utility of SoC versus the intervention in RH is determined.

**Methods:**

This is a multi-centre single-blinded randomised controlled trial (RHYME-RCT). First, at an eligibility visit, DBS sampling, to monitor drug concentrations in blood, and a 24-h ambulatory blood pressure measurement (24-h ABPM) are performed simultaneously. Patients with a daytime systolic blood pressure (SBP) > 135 and/or diastolic blood pressure (DBP) > 85 mmHg are randomised to SoC or intervention + SoC. The intervention is performed by the treating physician and includes information on drug concentrations and a comprehensive personalised feedback conversation with the use of a communication tool. The follow-up period is one year with visits at 3, 6 and 12 months randomisation and includes 24-h ABPM and DBS sampling.

**Discussion:**

This will be the first trial that focusses specifically on patients with RH without taking into account suspicion of non-adherence and it combines monitoring of AHD concentrations to identify non-adherence to AHDs with a comprehensive feedback to improve non-adherence. Furthermore, if this trial shows positive outcomes for the intervention it can be directly implemented in clinical practice, which would be a great improvement in the treatment of RH.

*Trial registration*. RHYME-RCT is registered in the Dutch Trial Register on 27/12/2017 (NTR6914) and can be found in the International Clinical Trials Registry Platform.

**Supplementary Information:**

The online version contains supplementary material available at 10.1186/s12872-023-03114-0.

## Introduction

Non-adherence to antihypertensive drugs (AHDs) is one of the most important causes of uncontrolled blood pressure. Uncontrolled blood pressure leads to high costs and suboptimal cardiovascular prevention [1–4]. When patients have uncontrolled blood pressures despite a medication regimen of AHDs from at least three classes including a diuretic, they are defined as having resistant hypertension. Resistant hypertension leads to more frequent outpatient clinic visits, more additional diagnostic tests and more hospitalizations because of a hypertensive emergency, stroke or myocardial infarction, and thus has a large individual and societal impact [2–4]. The majority of patients with resistant hypertension (estimated 40–60%) have a problem with adherence to their medication explaining their assumed resistance to therapy [5–10].

Identifying non-adherence and, more importantly, improving non-adherence is of major importance to reduce disease burden and costs[11–14]. For the identification step many tools are available and most have been investigated in previous interventions trials [15, 16]. The more reliable these identification methods become, the more invasive and costly they are. Electronic pill dispensers (also known as MEMS: Medication Events Monitoring Systems) are considered the gold standard for identification of non-adherence, but this method is not available on a large scale amongst all due to relatively high costs [2, 17, 18]. Recent research also showed the value of monitoring drug concentrations in blood or urine by (ultra-high) liquid chromatography-mass spectrometry (UHPLC-MS/MS) [19–21]. Although this is an objective and reliable method to determine non-adherence, the use in clinical practice is still limited [7, 9, 10, 22].

To improve the clinical usability of measurement of drug concentrations in blood, we have developed a dried blood spot (DBS) sampling method for AHDs. With this method, blood can easily be obtained by a finger or heel prick (similar to the diagnosis of inborn errors of metabolism in new-borns) [23]. DBS sampling enables immediate sampling in the office when non-adherence is expected without an additional visit to the blood sampling unit [23]. Using this method, non-adherence can be indisputably determined and (potentially undetectable) drug concentrations can be discussed with the patient. However, identification of non-adherent patients, will not directly improve non-adherence or blood pressure.

Therefore, consecutive steps are necessary. For the second step, improving non-adherence, behavioural interventions seem to be most successful [16]. Therefore, we developed a communication tool involving assessing barriers to adherence. Subsequently a personalised solution addressing the specific identified barrier of the patient can be realized. We think that the combination of monitoring drug concentrations with a behavioural intervention is a potential solution for solving the non-adherence issue.

Therefore, we aim to perform a randomised controlled trial to investigate whether showing patients the results of their drug concentration measurements combined with a behavioural change technique, is an effective intervention to improve adherence and thereby decreases the number of patients with assumed resistant hypertension.

## Methods

**Fig. 1:**
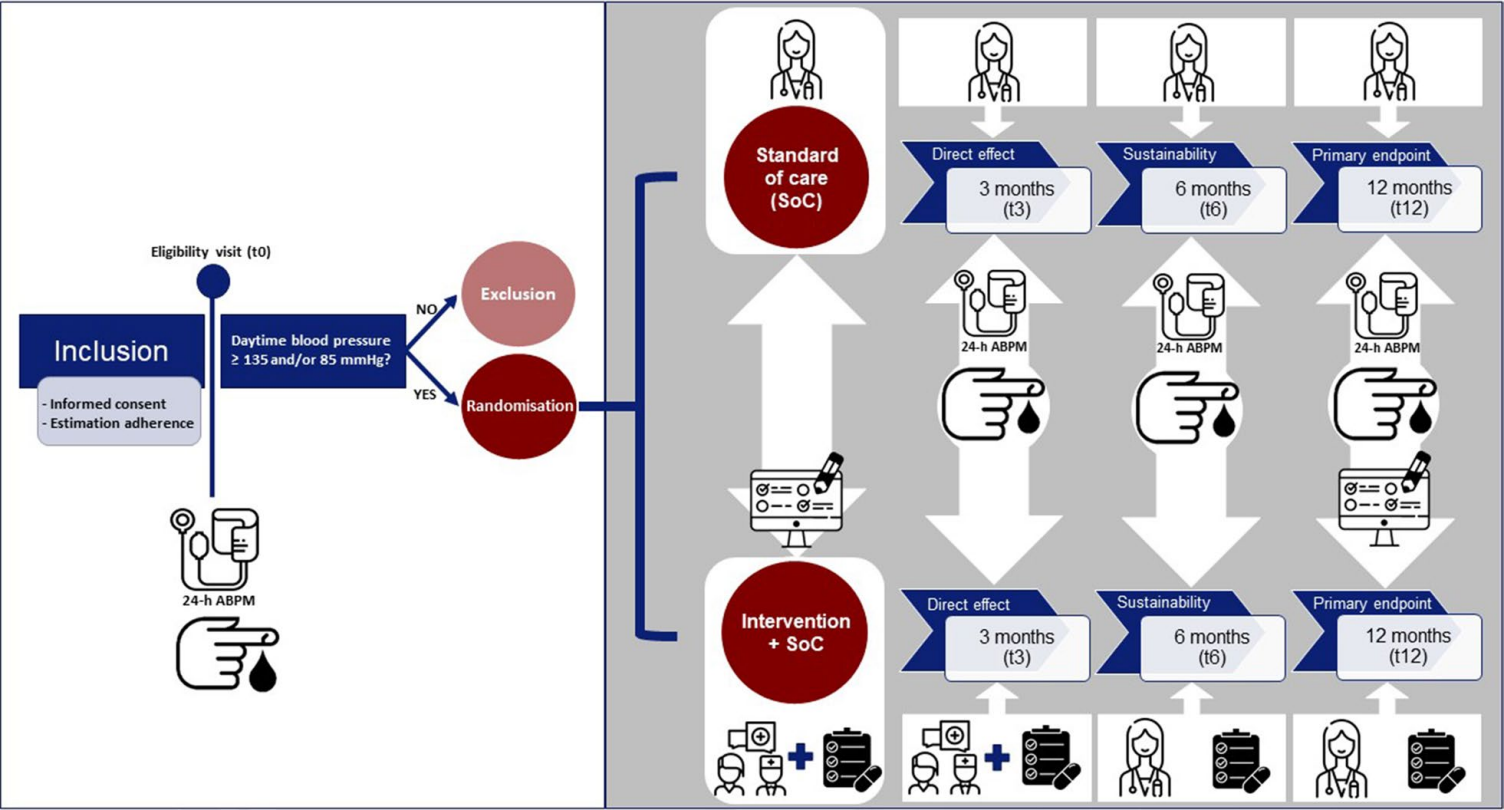
Study design RHYME-RCT trial to improve non-adherence to antihypertensive drugs and thereby blood pressure in patients with resistant hypertension

RHYME-RCT (ICTRP, NTR6914, https://trialsearch.who.int/Trial2.aspx?TrialID=NTR6914) is a randomised, multicentre, single-blinded, controlled trial to improve non-adherence to AHDs and reporting is based on the ESPACOMP Medication Adherence Reporting Guidelines (EMERGE) [24]. The study design and visit schedule are shown in Fig. 1 and Additonal file 1: Table S1.

### Subjects

#### Inclusion and exclusion criteria

Patients will be recruited from vascular, cardiology and nephrology outpatient clinics of 11 hospitals including two university hospitals. Patients are eligible for participation if they fulfill the criteria of resistant hypertension, use at least two AHDs for which DBS-analysis are available, are 18 years or older and are able to provide informed consent. Due to the large population of people orginating from Turkey in Rotterdam, the patient information leaflet is made available in both the Dutch and Turkish language.

Resistant hypertension is defined as having an office BP of > 140 mmHg (systolic) and/or 90 mmHg (diastolic) or, if available, a 24-h ambulatory blood pressure measurement (24-h ABPM) daytime BP of > 135 mmHg and/or 85 mmHg despite a medication regimen of AHDs in the maximal tolerable dose of at least three AHDs from different drug classes, including a diuretic, or at least four AHDs from different drug classes. In Table 1 the minimal drug dose needed at time of the inclusion and the lower limit of detection of each included drug are shown. DBS analysis includes the following AHDs and active metabolites: enalapril and enalaprilate, perindopril and perindoprilate, irbesartan, valsartan, losartan and losartan-carboxylic acid (losartan-CA), hydrochlorothiazide, bumetanide, spironolactone and canrenone, amlodipine, barnidipine, nifedipine, metoprolol and doxazosin [25, 26].

**Table 1:** Overview antihypertensive drugs included in the RHYME-RCT trial

Antihypertensive drug [metabolite]	Minimal drug dose for inclusion (mg)	LLOD (μg/L) [25]
Amlodipine	5	17.1
Barnidipine	10	2.1
Bumetanide	1	4.0
Doxazosin	4	18.1
Enalapril	20	0.4
[Enalaprilat]	–	1.1
Hydrochlorothiazide	12.5	40.2
Irbesartan	150	7.7
Losartan	50	1.7
[Losartan-CA]	–	2.6
Metoprolol	50 CR or 25 mg two times daily (normal release)	0
Nifedipine	30	3.5
Perindopril	4	0.7
[Perindoprilat]	–	1.3
Spironolactone	12.5	5.2
[Canrenone]	–	26.8
Valsartan	80	21.3

*CA* carboxylic acid; *CR* controlled release, *LLOD* lower limit of detection

Patients are excluded from participation if they are not able to give informed consent, have end-stage kidney disease (eGFR < 15 ml/min/m^2^), insufficient understanding of the Dutch or Turkish language or if secondary forms of hypertension are expected but have not been excluded. Patients with secondary forms of hypertension primarily treated with AHDs such as primary aldosteronism caused by bilateral hyperplasia can be eligible for participating in the trial.

This study is approved by the Institutional Review Board (IRB) of the Erasmus MC, University Medical Centre, Rotterdam, the Netherlands (MEC-2018–027).

#### Withdrawal

Patients are allowed to withdraw from the trial at any time without a statement of reason. However, when a reason is given by the patient, this will be noted. Also, investigators can decide to withdraw a subject from the study for urgent medical reasons. Data from withdrawn patients will be included if possible. Patients that withdraw before reaching 12 months follow-up will be replaced.

### Study design

Eligible patients are informed about the trial by the treating physician. Patients are told that with the fingerprick drug concentrations in blood are measured. However, the term adherence will not be mentioned to the patients and is not included in the patient information leaflet. If the patient is interested in participation, written information will be provided. After at least one week the patient is called by the researchers, to answer patients’ questions regarding study participation. When verbal consent is given, an eligibility visit is scheduled. At this eligibility visit, called t0, written informed consent will be obtained, a 24-h ABPM is performed and simultaneously a finger prick for therapeutic drug monitoring (TDM) is performed. Medication is not standardised prior to inclusion.

Based on the results of the 24-h ABPM during t0, patients are either excluded or randomised to a ‘personalised feedback conversation’ (intervention) added to standard of care (SoC) or SoC alone (Fig. 1).

After randomisation the treating physician is asked to estimate if the patient was adherent to AHDs or not, to determine if there is any bias in selecting patients. The selection of patients is based on blood pressure and thereby resistant hypertension, but physicians can potentially approach only patients they expected to be non-adherent. The adherence estimation is always performed before outcomes on non-adherence are reported (applicable for the intervention + SoC arm).

#### Randomisation

The hospitals participating in the trial are divided into strata. For each stratum random block randomisation is performed in blocks of 4–6. Within these blocks patients are randomly assigned to either the SoC alone or intervention + SoC arm in a 1:1 ratio. Results on randomisation are communicated to the health care providers.

#### Blinding

The measurements performed to determine non-adherence are only reported to the health care providers when patients ware randomised to the intervention + SoC arm. Measurements from the control arm are only available after completion or withdrawal of the study. Also, the laboratory that performed the measurements are not aware of the randomisation or which ID belonged to which patient. Only the coordinating researcher is not blinded and thereby is able to report the results from the intervention + SoC arm patients to the right physician. The coordinating researcher does not perform the actual intervention.

#### Standard of care (SoC) alone

SoC includes visits at 3 (t3), 6 (t6), and 12 (t12) months follow-up, with preferably a 24-h ABPM, and otherwise an automated office blood pressure (AOBP) combined with DBS sampling. In the SoC arm the results on drug concentrations are not reported during the study and are only used for resarch purposes.

The results of the blood pressure measurements are available for all healthcare providers. If deemed necessary by the treating physician it is allowed to schedule extra appointments outside the research visits. Also, it is allowed to make changes to the AHD treatment during every visit, without restriction with regard to the choice or dose of drugs. These changes are registered at every visit. If a patient switched to AHDs that are not included in the TDM method, only blood pressure measurements are included in the analysis.

Patients are allowed to make life style changes during the trial. These life style changes can possibly influence the adherence outcome and is therefore taken into account in our power calculation.

#### Intervention + SoC

The intervention can be described as a ‘comprehensive personalised feedback conversation’ added to SoC, and consists of a three-step method [27]. For this comprehensive converstation extra consultation time was planned for phycisians that usually only have 10-min consultations. When patients are going to a nurse practioner for blood pressure control, no extra time will be planned while they already have 30-min consultations.

First, results of the AHD concentrations measured with UPLC-MS/MS are reported back to the healthcare provider.

In the second step of the intervention, the treating physician or nurse practitioner have to relay the findings of the drug concentrations in a non-accusatory way. To support and guide exploration of reasons for non-adherence a communication tool is developed by a sociologist and psychologist as previously discribed by Peeters et al [27]. By examining the reasons for non-adherence, tailored solutions can be agreed upon. Therefore, in the final step, based on the individualised determinants of adherence and potential solutions, a plan is made to improve adherence to AHDs.

All health care providers have to follow a training beforehand to improve their communication skills. This training includes a 90 min lecture on the theory around adherence and how to improve this with communication. After the lecture, participants have one hour to practice the feedback conversation with one another, coached by a sociologist or psychologist. Also, a digitial version of the training is available as a booster.

Patients in the intervention + SoC arm receive personalised feedback at t0 and t3. At t6 and t12 results on drug concentrations are still reported to the treating health care providers, but not communicated to the patient to determine sustainability of the earlier intervention (Fig. 1).

### Methods of measurement

#### Blood pressure measurement

24-h blood pressure is recorded with the available ABPM device on site. All devices have to be approved for use in clinical practice. Measurements are performed with the device attached to the non-dominant arm if possible. Patients are instructed to relax their arm during the measurements and to pursue their normal daily activities. During the 24-ABPM patients are also asked to write down physical and stressful activities in a diary, as well as their true sleeping times. Depending on the hospital, BP is measured at a 20-min or 30-min interval during daytime and a 30-min or 60-min interval at night-time. Measurements are included if > 50% of the 24-h measurements was successful which includes at least 20 valid awake measurements and 7 valid asleep measurements [28, 29]. If a patient is eligible for the study because of a 24-h ABPM ≥ 135 and/or 85 mmHg measured for clinical purposes and the fingerprick is obtained within 4 weeks after this measurement, it is a allowed to use this ABPM measurement as inclusion criterion. However, an AOBP has to be performed at time of the fingerprick and needs to be ≥ 140 and/or 90 mmHg. If a patient does not want to do a 24-ABPM during the eligibility visit, they are excluded from participation. At all the follow-up visits, the 24-h ABPM can be replaced with a 30-min AOBP upon reasonable request of the patient as stated before.

#### Adherence

Two validated UPLC-MS/MS methods combined with a DBS sampling method are used to determine non-adherence to the aforementioned AHDs up until 24 h after intake of the drug [25, 26].

Sampling has to be performed by trained healthcare providers or researchers, but on occasion also by study subjects themselves after an oral and written instruction. Sampling is performed by means of a blood lancet (BD microtainer 2.0 mm × 1.5 mm) and filter papers (Whatman™, protein saver, 903 card). Five drops of blood need to be sampled per patient per time point of which at least one is needed for analysis. Each drop sampled on the paper has to be at least 6 mm in diameter to assure accurate measurement. The patient is asked to estimate the time of intake of the AHDs and time of sampling will be registered by the researchers. Samples are sent by mail in a paper envelope containing a lab form with information on the sample and a moisture absorber bag. Measurements of drug concentrations in the lab are performed every two weeks to assure minimal degradation of the drugs. Furthermore, outcomes of the drug concentrations have to be reported in time to the health care providers of the patients randomised to the intervention + SoC arm.

The adherence of patients is determined by means of the absence [< lower limit of detection (LLOD)] or presence of all the expected drugs in blood (Table 1). Expected drugs are drugs that were prescribed and measurable with the previously validated DBS method [25, 26]. When none of the expected drugs are detected in the patient’s blood, the patient is scored as non-adherent. Furthermore, when one or more, but not all drugs are absent from blood, the patient is scored as partially non-adherent. When all expected drugs are found, the patient will be considered adherent.

#### Questionnaires

At t = 0 months and t = 12 months patients are asked to fill-in 4 questionnaires. These questionnaires are send either by e-mail or, when requested, by mail. These questionnaires include the beliefs about medicine questionnaire (BMQ), EuroQol-5D-5L (EQ-5D-5L) to determine health-related quality of life, iMTA productivity cost questionnaire (PCQ) and medical consumption questionnaire (MCQ) for economic evaluation which are adjusted for use in our trial [30–32].

Finally, at 12 months follow-up patients are asked to fill in an evaluation questionnaire with regard to the overall experience of the trial.

#### Other study parameters and assessments

At t0 and t12 patient measurement values and available laboratory values are collected (Additional file 1: Table S1). This includes relevant comorbidities, mainly related to cardiovascular diseases, which are derived from the patient record system. Furthermore, serious adverse events (SAEs) are registered troughout the trial up and till four weeks after the last measurement (Additional file 1: Table S1). SAEs are actively reported to the coordinating researcher. Adverse events related to blood pressure are collected from the patient record system.

### Study endpoints

#### Primary objective

Our primary objective is to determine whether an intervention that consists of monitoring AHD concentrations combined with personalised feedback added to SoC leads to a larger decrease in the prevalence of RH after 12 months of follow-up than SoC alone.

#### Secondary objectives

Secondary endpoints include the effect of the intervention on the proportion of patients adherent to AHDs and patients fulfilling the definition of RH over time including baseline (t0), t3, t6 and t12.

Furthermore, the number of required AHDs as well as the defined daily dose (DDD) is calculated at t0 and t12 in both arms to determine if there is a difference over time between the arms.

Lastly, the cost-utility of the intervention vs standard of care in resistant hypertension is determined.

### Data analysis

#### Sample size calculation

The sample size calculation is based on the expected improvements in the prevalence of RH due to improvements in adherence. It is assumed that around 50% of eligible patients is (partially) non-adherent at the moment of randomisation. Based on several adherence trials, we expect a decrease of non-adherence by a relative 40% and 20% in those assigned to the intervention + SoC and SoC alone, and, hence, the expected prevalence of RH at 12 months is expected to be 80% (intervention + SoC) and 90% (SoC alone), respectively [33–35] . A total of 2*196 = 392 patients is required after 12 months of follow-up to demonstrate this difference with β = 0.8 (power = 80%) and a two-sided α = 0.05.

#### Interim analysis

An interim analysis will be performed with data from the trial to check the assumptions made in the sample size calculation. For this at least 25 patients have to be included in both arms that reached three months follow-up. We expect to find a larger differerence at this time point due to the shorter follow-up time, as compared to results after 12 months follow-up when the effect of the intervention is expected to be slightly waned of.

#### Statistical analysis

Continous variables with a normal distribution are described as mean value ± one standard deviation (SD), and otherwise as median value (25th–75th percentile). Normality is tested by the Shapiro–Wilk test. Categorical variables are described as numbers and percentages. Within group changes in percentage non-adherence and RH from t0 to t12 are evaluated by McNemar tests. We use a Chi^2^-test to study change-differences in non-adherence and RH between patients randomised to the intervention + SoC versus SoC alone.

The defined daily dose (DDD) is the assumed average maintenance dose of a drug used for its main indication in adults [36]. These averages are used to determine how many times the DDD for each individual drug is used. Subsequently, the averages of these DDDs are calculated for t0 and t12 for individual patients.

Differences in blood pressure and DDDs over time within the same arm are tested by means of a paired sample t-test and differences between patients randomised to the intervention + SoC versus SoC alone with a independent samples t-test.

We will perform an intention-to-treat analysis, including all randomised patients, as well as a per-protocol analysis, including the patients with available measurements at t0 and t12, and one of t3 and t6.

Data from the questionnaires are used for the cost-effectiveness and cost-utility analysis of the intervention. A cost-utility analysis will be performed with quality adjusted life years (QALY) for a lifelong period. The QALYs will be calculated based on the EQ-5D-5L summary score. Cost-effectiveness from a healthcare perspective will be calculated for a 1-year period using the number of patients fulfilling the definition of resistant hypertension after 12 months (the primary outcome measure).

All p-values are two-sided, and a value of < 0.05 is considered statistically significant.

We used the SPSS version 24.0 for Windows (IBM Corp, Armonk, NY), and GraphPad Prism 9.3 software (GraphPad Software, La Jolla, CA) for analysis.

## Discussion

This will be the first trial that focusses specifically on patients with RH and combines measurement of drug concentrations to identify non-adherence to AHDs, with a comprehensive feedback to the patients to improve non-adherence. Measurement of drug concentrations is not hampered by the availability of places for phlebotomy, as DBS sampling can be performed by anyone at any time with the right training and materials. Also, patients are selected based on their blood pressure and number of prescribed AHDs instead of based on the adherence estimation of physicians. Hereby, including patients that otherwise would not be recognized as being non-adherent. Lastly, if this trial shows positive outcomes for the intervention it can be directly implemented in clinical practice, which would be a great improvement in the treatment of RH.

However, there are some possible problems that can occur due to the study design. First, patients are obliged to do a 24-h ABPM to eliminate white coat hypertension. This blood pressure measuring method is the most reliable to establish true hypertension, but not patient friendly, as it often interferes with planned daily activities and the ability to sleep [37, 38]. Therefore, this could lead to the exclusion of a specific group of patients that potentially do not have true resistant hypertension [39]. We tried to make the trial as patient friendly as possible, to include, preferably, daytime measurements or AOBP as alternatives for the 24-ABPM if a patient tends to drop-out because of the ABPM.

Second, not all AHDs could be included in the UPLC-MS/MS method since extensive validation is necessary before use in clinical practice [25]. For inclusion patients have to use at least two AHDs that can be measured with our method, but it is not obliged to only use AHDs that can be measured. As a result, there is a considerable chance that we are not able to measure all used antihypertensive drugs, and potentially miss non-adherence to certain AHDs. However, the importance of adherence will be mentioned during feedback conversations thereby improving non-adherence to AHDs we are not able to measure.

Third, we are aware of the fact that our SoC arm is not actual standard of care in clinical practice. In clinical practice four 24-h ABPM in one year for every patient, is not performed nor recommended by the guidelines [29, 40]. However, as mentioned before, the most accurate blood pressure measuring method available [37]. Furthermore, to compare both arms, DBS sampling will be performed in both trial arms, which is also not standard of care. These additional measurements can possibly influence the outcome, but we anticipated this and therefore made some corrections for this phenomenon in sample size calculation.

In conclusion, this is a unique trial with innovative but easy to use technologies to both identify and improve non-adherence to antihypertensive drugs in a population where treatment options are limited. Also, clinical implementation can easily be carried out in hospitals but also at GPs offices, when the intervention is found to be effective.

## Supplementary Information

**Additional file 1**: **Table S1**. Visit schedule RHYME-RCT trial.

## Data Availability

The data that support the findings of this study are available upon reasonable request from the PI, [JV] following a 12 months embargo from the date of publication to publish additional results from the study (in accordance with our statement at www.zorggegevens.nl).

## Abbreviations

24-h ABPM: 24-hour ambulatory blood pressure measurement
AHDs: Antihypertensive drugs
BMQ: Beliefs about medicine questionnaire
DBP: Diastolic blood pressure
LLOD: Lower limit of detection
MCQ: Medical consumption questionnaire
PCQ: Productivity cost questionnaire
RH: Resistant hypertension
SBP: Systolic blood pressure
SoC: Standard of care
TDM: Therapeutic drug monitoring
UHPLC-MS/MS: Ultra-high performance liquid-chromatography-mass spectrometry
