# Supplementary material for: The effect of combining therapeutic drug monitoring of antihypertensive drugs with personalised feedback on adherence and resistant hypertension: the (RHYME-RCT) trial protocol of a multi-centre randomised controlled trial
Source: BMC Cardiovasc Disord. 2023 Feb 14;23:87. doi: 10.1186/s12872-023-03114-0 (PMC9926861; doi:10.1186/s12872-023-03114-0)
Supplement: Supplementary file 1 — Additional file 1: Table S1. Visit schedule RHYME-RCT trial. [file 12872_2023_3114_MOESM1_ESM.docx]

Supplemental material Table 1 Visit schedule RHYME-RCT trial

| Visit | V-3 | V-2 | V-1 | V0 | V1* | V2 | V3 | V4 | V5 | V6 | V7 |
| --- | --- | --- | --- | --- | --- | --- | --- | --- | --- | --- | --- |
| Visit Description | Scree-ning | Hand over PIL | Contact patient | First 24-h ABPM and finger prick | Intervention (or standard of care) | 3 Mo 24-h ABPM and finger prick | 3 Mo visit | 6 Mo 24-h ABPM and finger prick | 6 Mo visit | 12 Mo 24-h ABPM and finger prick | 12 Mo visit |
| Visit Window (days) | NA | NA | 14 post V-2 | 28 post V-1  **(t=0)** | > 14 days post V0  **(t=0)** | 75±14  **(t=3)** | > 14 days post V2  **(t=3)** | 165±14  **(t=6)** | > 14 days post V4  **(t=6)** | 360±14  **(t=12)** | > 14 days post V6 **(t=12)** |
| Office BP | X |  |  |  |  |  |  |  |  |  |  |
| Review medication | X |  |  |  |  |  |  |  |  |  |  |
| Inform patient |  | X | O/X | X |  |  |  |  |  |  |  |
| Written informed consent |  |  |  | X |  |  |  |  |  |  |  |
| 24-hour Ambulatory BP |  |  |  | X |  | X |  | X |  | X |  |
| Finger prick |  |  |  | X |  | X |  | X |  | X |  |
| Printed medication overview or digital CRF |  |  |  | X |  |  |  |  |  | X |  |
| Patient measurement values |  |  |  | X |  |  |  |  |  | X |  |
| Lab values (including creat, eGFR, proteinuria) |  |  |  | X |  |  |  |  |  | X |  |
| Comorbidities |  |  |  | X |  |  |  |  |  | X |  |
| Randomisation* |  |  |  | O/X* |  |  |  |  |  |  |  |
| Feedback conversation |  |  |  |  | X |  | X |  |  |  |  |
| Results DBS (only intervention group) |  |  |  |  | X |  | X |  | X |  | X |
| Questionnaires patient |  |  |  |  | O |  |  |  |  |  | O |
| Estimation adherence |  |  |  | X |  |  |  |  |  |  |  |
| Document changes in medication |  |  |  |  | X |  | X |  | X |  | X |
| Adverse events |  |  |  |  | X |  | X |  | X |  | X |
| Protocol deviations |  |  |  | X | X | X | X | X | X | X | X |
| Drop out |  |  |  |  | X | X | X | X | X | X | X |

*BP: Blood Pressure, Mo = Month; O = coordinating researcher will carry out this activity; CRF = Case Report Form; PIL = Patient Information Leaflet; V = Visit; t = Time, ABPM = ambulatory blood pressure measurement*
*Only patients with the following 24-hour blood pressure measurement are eligible to enter RHYME-RCT and can continue to visit V1:
 * Day average systolic blood pressure > 135 mmHg OR Day average diastolic blood pressure > 85 mmHg
 OR * Day average systolic blood pressure > 135 mmHg AND Average diastolic blood pressure > 85 mmHg
The 24-hour blood pressure recording has to have at least 50% of the expected measurements with 20 valid awake measurements.*
